# Population-level plasticity in foraging behavior of western gulls (*Larus occidentalis*)

**DOI:** 10.1186/s40462-017-0118-9

**Published:** 2017-12-19

**Authors:** Scott A. Shaffer, Sue Cockerham, Pete Warzybok, Russell W. Bradley, Jaime Jahncke, Corey A. Clatterbuck, Magali Lucia, Jennifer A. Jelincic, Anne L. Cassell, Emma C. Kelsey, Josh Adams

**Affiliations:** 1 Department of Biological Sciences, San José State University, San Jose, CA 95192-0100 USA; 2 University of California, Institute of Marine Sciences, Santa Cruz, CA USA; 3 Point Blue Conservation Science, Petaluma, CA USA; 4 Biology Department, San Diego State University, San Diego, CA USA; 5 Western Ecological Research Center, U.S. Geological Survey, Santa Cruz Field Station, Santa Cruz, CA USA

**Keywords:** Fidelity index, GPS tracking, Habitat use, Time activity budgets, Urban habitats

## Abstract

**Background:**

Plasticity in foraging behavior among individuals, or across populations may reduce competition. As a generalist carnivore, western gulls (*Larus occidentalis*) consume a wide range of marine and terrestrial foods. However, the foraging patterns and habitat selection (ocean or land) of western gulls is not well understood, despite their ubiquity in coastal California. Here, we used GPS loggers to compare the foraging behavior and habitat use of western gulls breeding at two island colonies in central California.

**Results:**

Gulls from offshore Southeast Farallon Island (SFI; *n* = 41 gulls) conducted more oceanic trips (*n* = 90) of shorter duration (3.8 ± 3.3 SD hours) and distance (27.1 ± 20.3 km) than trips to the mainland (*n* = 41) which were nearly 4 times longer and 2 times farther away. In contrast, gulls from coastal Año Nuevo Island (ANI; *n* = 20 gulls) foraged at sites on land more frequently (*n* = 103) but trip durations (3.6 ± 2.4 h) and distances (20.8 ± 9.4 km) did not differ significantly from oceanic trips (*n* = 42) where trip durations were only slightly shorter (2.9 ± 2.7 h) and equidistant (20.6 ± 12.1 km). Gulls from both colonies visited more sites while foraging at sea but spent significantly longer (3–5 times) durations at each site visited on land. Foraging at sea was also more random compared to foraging trips over land where gulls from both colonies visited the same sites on multiple trips. The total home range of gulls from SFI (14,230 km^2^) was 4.5 times larger than that of gulls from ANI, consistent with greater resource competition resulting from a larger abundance of seabirds at SFI.

**Conclusions:**

Population-level plasticity in foraging behavior was evident and dependent on habitat type. In addition, gulls from SFI were away foraging longer than gulls from ANI (22% vs. 7.5%, respectively), which impacts the defense of territories and attempts at nest predation by conspecifics. Our results can be used to explain lower chick productivity at SFI, and can provide insight into increased gull activity in urban areas.

**Electronic supplementary material:**

The online version of this article (10.1186/s40462-017-0118-9) contains supplementary material, which is available to authorized users.

## Background

Links between resource availability and foraging behavior result in decisions at the individual level that can influence breeding success and ultimately population dynamics [1, 2]. Variation in foraging behavior among individuals (i.e. ‘individual specialization’) can reduce niche overlap within a population, thereby decreasing competition [3, 4]. Growing evidence demonstrates that individual specialization within a population, or even across populations within a species, is perhaps more common than previously thought [5–10]. This variation in foraging behavior may ultimately facilitate adaptation to changing environmental conditions, especially for species that are considered generalists.

Collectively, *Larus spp.* gulls are regarded as generalist carnivores, known for their ability to exploit both marine and terrestrial resources. They nest in a variety of habitats [11–13] and consume both marine and terrestrial foods that can vary with breeding stage and availability [14–18]. More recent studies using tracking technologies indicate that gulls employ a range of foraging strategies to reduce intraspecific competition through spatial segregation and variations in diet [9, 19–21]. Understanding this variation is essential because it can influence resource management decisions [22], such as evaluating interactions between wind turbines and gull populations [23–25], or managing fisheries discards [26–28].

Despite recent information on the foraging behavior and movement ecology of gulls, there is a lack of information about the approximately 22 gull species that frequent the Pacific Ocean. Excluding kittiwakes *Rissa* spp., which have been studied during breeding and non-breeding phases [29–31], we are aware of only three Pacific species, glaucous-winged (*Larus glaucescens*), black-tailed (*L. crassirostris*), and swallow-tailed (*Creagrus furcatus*) gulls, where detailed movements and distribution patterns of individual gulls have been reported [32–34]. Moreover, only one study [34] included data from multiple gull populations.

Western gulls (*L. occidentalis*) range throughout temperate coastal zones of the eastern North Pacific, with the largest breeding colonies in central and southern California [35]. Although considered marine predators, western gulls are known to utilize waste recovery landfills and other refuse sites as food sources while breeding [14, 36, 37]. However, despite being one of the most conspicuous gull species in California, foraging behavior and habitat use patterns of western gulls (e.g. basic foraging metrics, activity budgets and effort, patterns of habitat use and distribution while foraging) are poorly understood (but see [36]). Thus, it is unclear how foraging behavior varies at the individual- or population-level, or how habitat influences foraging activity in this gull species. This information could help explain the shift in western gull diets observed throughout the past century [38] and why western gulls remain ubiquitous throughout their range, despite human impacts on coastal resources [39, 40].

In this study, we compared the foraging behavior of two western gull populations in central California using GPS loggers deployed on breeding individuals during three consecutive years. We tested whether 1) there were differences in specific foraging behaviors and activity patterns between and within gull populations, 2) activity patterns differed for gulls that foraged in contrasting environments (i.e. marine vs. terrestrial), and 3) colony proximity to the coast influenced the frequency of habitat choice in gulls from each population. Because we tracked individual gulls across multiple foraging trips, we also tested whether gulls exhibited repeatability in foraging distribution and if destinations differed by habitat type. Ultimately, our characterization of foraging behavior and activity allowed us to evaluate colony attendance patterns with implications for explaining differences in reproductive success between two gull populations.

## Methods

### Field sites and breeding colonies

We studied western gulls at two central California breeding colonies: Southeast Farallon Island (SFI: 37.697°N, 123.001°W), within the Farallon Islands National Wildlife Refuge, located 45 km off the coast of San Francisco, California, USA and at Año Nuevo Island (ANI: 37.108°N, 122.337°W) located 1 km from shore in Año Nuevo State Park, San Mateo County, California, USA. SFI is 31 ha and hosts 13 breeding species of seabirds, including the largest western gull colony in California (breeding population of 10,000 to 25,000 gulls [41]). In contrast, ANI is only 4 ha, hosts 7 breeding species of seabirds, and has a breeding gull population of 700–1000 pairs [42]. Nests on both islands have rocky or sandy (ANI) substrate with low ground cover; the two islands are separated by 88 km of open ocean.

### Logger deployments and sampling

Western gulls were studied during the incubation phase of breeding between May and June of 2013–2015 (see Additional file 1:Table S1 for details). A total of 52 adults were captured at SFI and 37 adults were captured at ANI. All gulls were breeding individuals captured with a noose mat or single foot snare at their nest (all study nests had 2–3 eggs). Western gulls are sexually size dimorphic (males are ca. 20% larger; [35]), and sex can generally be ascribed between mated pairs by observation and comparison in the field. However, there is overlap in body size measurements (e.g. body mass, bill length, etc.); therefore, in the absence of molecular sexing, indices of foraging behavior for both sexes were combined in the present study.

Upon capture and removal from foot snares, gulls were placed into a pillowcase and weighed using a spring balance (±20 g; Pesola scales, Schindellegi, Switzerland). Birds were ringed with a stainless steel USFWS identification band and equipped with a small, GPS data logger attached at the base (posterior of preen gland) of 3–4 central tail feathers using Tesa tape (Tesa Tape Inc., Charlotte, NC). GPS loggers (either 20-g igotU GT-120 or 32-g igotU GT-600, Mobile Action Technology, Taiwan) recorded a location every 1–2 min with a spatial accuracy of 3–4 m (manufacturer specifications). To make GPS loggers as light as possible, each unit was removed from its original plastic case and encapsulated in lighter adhesive-lined heat shrink tubing to prevent water intrusion. Larger GPS units (i.e. GT 600) were used in less than 10% of deployments and were only deployed on gulls weighing in excess of 1100 g. Thus, in total, gulls were equipped with a package that weighed between 2 and 3% of total gull body mass.

Gulls were recaptured after 4–8 days at their nests using the same methods described earlier. GPS loggers were removed, body mass measured again, and linear dimensions of the exposed culmen, tarsus, and skull (i.e. combined head & bill length) were measured with calipers (±0.5 mm). Total handling time for all procedures was 5–15 min. Gulls often regurgitated when captured, so opportunistic diet samples were also collected in the field and stored at −20 °C until dietary analyses were conducted for a separate study [37].

### Analysis of tracking data and characterization of movement patterns

For each gull, the complete set of tracking data were quality controlled (QC) by 1) removing data that preceded logger deployment and followed logger recovery and 2) using only complete trips with a clear beginning and end from a central place (i.e. colony). Furthermore, all complete tracks were filtered using a backward–forward iterative speed filter [43] based on a maximum travel speed of 70 km h^−1^ [44, 45]. Location filtering resulted in the removal of less than 0.01% of all QC positional fixes (*N* = 325,171 locations after filtering). All subsequent data analyses were performed on interpolated tracks with two minute intervals to remove gaps between GPS locations (mean of max gap interval per track 3.30 ± 2.45 min, *N* = 276 tracks).

Because gulls occasionally made short trips off shore of each island (presumably to drink, rest, etc.), we only considered trips that were greater than 30 min in duration and farther than 1 km away from the breeding colony. Hence, a 1-km buffer was used to establish all departure (outbound) and return (inbound) times based on travel outside the buffer. The duration of a single trip was measured as the total time outside the buffer zone and nesting intervals were determined as the total time inside the buffer, between foraging trips, when gulls were present in the colony (based on location data and/or visual confirmation). The total distance traveled by each gull was the cumulative distance between all consecutive locations along a trackline for a single trip and the maximum range (i.e. distance) was the farthest location away from the colony during a trip. All track analyses were performed with purpose-built routines created in MATLAB R2016b (The Mathworks, Natick, MA) using functions from the *Mapping* and *Statistics and Machine Learning* toolboxes.

### Analysis of trip metrics and spatial distributions

For each trip away from the colony, we first determined whether gulls traveled to the coast or remained offshore. Each trackline was projected onto high resolution coastline data (Global Self-consistent, Hierarchical, High-resolution Shoreline Database; gshhs v.2.3.5; [46]) and if a gull’s flight path crossed over land, it was classified as a ‘land’ trip. For each trip, we determined the total time spent over land vs. ocean (i.e. in minutes and as percentage of trip duration) and direction of travel from the colony. Home ranges for each populaton were calculated using minimum convex polygons (MCP) around all tracks from a given colony using the *convhull* function in MATLAB. The area (in km^2^) and overlap between MCP’s for each population were calculated [7].

### Characterization of behavior and time activity budgets

Behavioral state was characterized by determining whether gulls were in transit or stationary along a trackline by calculating the travel speed between GPS positional fixes. The stationary events were considered potential foraging opportunities, whether at sea or over land. Gulls were considered stationary when travel speeds were <6 km h^−1^ for a minimum of two consecutive GPS fixes (Additional file 2: Figure S1). Stationary events for land trips were further delineated based on a minimum of 5 min within 0.5-km radius. This additional criterion considered gull movement within a discrete land location resulting in multiple takeoffs and landings within the 0.5-km radius (see below). For each trip, we quantified the 1) number and duration of each stationary event, 2) spatial extent and location of the stationary event, 3) the geographic center of each stationary event, and 4) the distance between each successive stationary event. Because stationary events typically included multiple positional fixes as gulls paddled or walked around, or took off and landed during a given stationary event, we calculated the area (in m^2^) of the minimum convex polygon encompassing all locations where gulls had minimal movement during a stationary event (Additional file 3: Figure S2). The centroid of each polygon was considered the geographic location of the stationary event and the distance (in km) between stationary events was calculated.

Stationary events on land by gulls were further investigated using Google Earth (Google Earth Pro v.7.1.7.2600) to identify and delineate different habitats visited based on six categories 1) urban (e.g. buildings, parks, and schools in urban areas), 2) piers in urban area, 3) freshwater lakes within parks, 4) landfills and food recycling centers, 5) bluffs along the coast, and 6) beaches river outflows along coast. Every gull that visited one of these habitats was identified and the total number of visits by all gulls was determined.

For comparison of behavioral patterns associated with habitat, all metrics (e.g. number of stationary events, distance between each event, etc.) for land trips quantified only those portions of a trip when gulls were traveling/stationary while over land.

We also established time activity budgets for each gull based upon the proportion of time spent away from the colony versus the proportion of time in the colony during the logger deployment period. Overall, this information allowed us to compare the activity patterns of gulls by population and habitat type (classified as ocean or land) of foraging trips.

### Comparative patterns of habitat use

To compare foraging patterns of gulls from each population and habitat type (i.e. ocean vs. land), we calculated a Fidelity Index (FI) based on a model proposed by [47]. Firstly, the distance (in km) and net angular displacement (in degrees) of the farthest location from a breeding colony for each foraging trip was averaged (using circular statistics for angle means) for all gulls based on colony origin and habitat type. Then, the normalized difference in distance between an individual trip (*dist*_*i*_) and colony/habitat mean (*dist*_*c*_) was summed with the normalized difference in net angular displacement between an individual trip (*angle*_*i*_) and colony/habitat mean (*angle*_*c*_) using the formula:

$$ FI=2\times \left[\left({dist}_i-{dist}_c\right)\div {dist}_i\right]+\left[\left({angle}_i-{angle}_c\right)\div 90\right] $$

FI values ranged between 0 and 4, where FI = 0 for an individual trip was considered the *most similar* to the colony/habitat mean (i.e. high fidelity) and FI = 4 considered the least similar to the colony/habitat mean (i.e. low fidelity). A gull traveling the same maximum distance away but in opposite direction as the colony/habitat mean would have a FI = 2, an equivalent weighting to a gull traveling half the distance with a net angular displacement of 90° (sensu [47]).

### Statistical analyses

Linear mixed-effects models (LME; *fitlme* function in MATLAB R2016b) were used to compare trip metrics and behavioral patterns of gulls according to population (SFI or ANI) and by habitat type (ocean or land) as fixed factors, and bird ID as a random factor to account for the different number of trips per bird. Proportional data were transformed using a logit function and compared using linear mixed-effects models. Restricted Maximum Likelihood (REML) estimations were used and Type 3 Sums of Squares were compared to account for an unbalanced design. Prior to using LME, all non-normal data (maximum range and distance traveled) were transformed using a log transformation. All data presented in figures or text are shown as untransformed values and as mean ± SD unless otherwise stated. Statistical significance was evaluated at *P* ≤ 0.05.

## Results

### Logger deployments and recovery

A total of 89 western gulls from both colonies combined were captured and equipped with GPS data loggers during the study period. Overall, 76 gulls (85%) were recaptured and 69 loggers (78%) were recovered (see Table S1 for yearly deployments by population). Of the loggers recovered, 61 (88%) yielded viable data resulting in a total of 276 foraging trips. Eighty-six of the eighty-nine gulls originally captured (97%) continued breeding beyond the study period in each year. The gulls that abandoned their breeding effort had the contents of their nests predated and thus departed before recapture was possible. Gulls recaptured without a logger either pulled feathers or weakened the adhesive tape attachment, allowing the logger to fall off prematurely.

### Comparative trip patterns

**Fig. 1:**
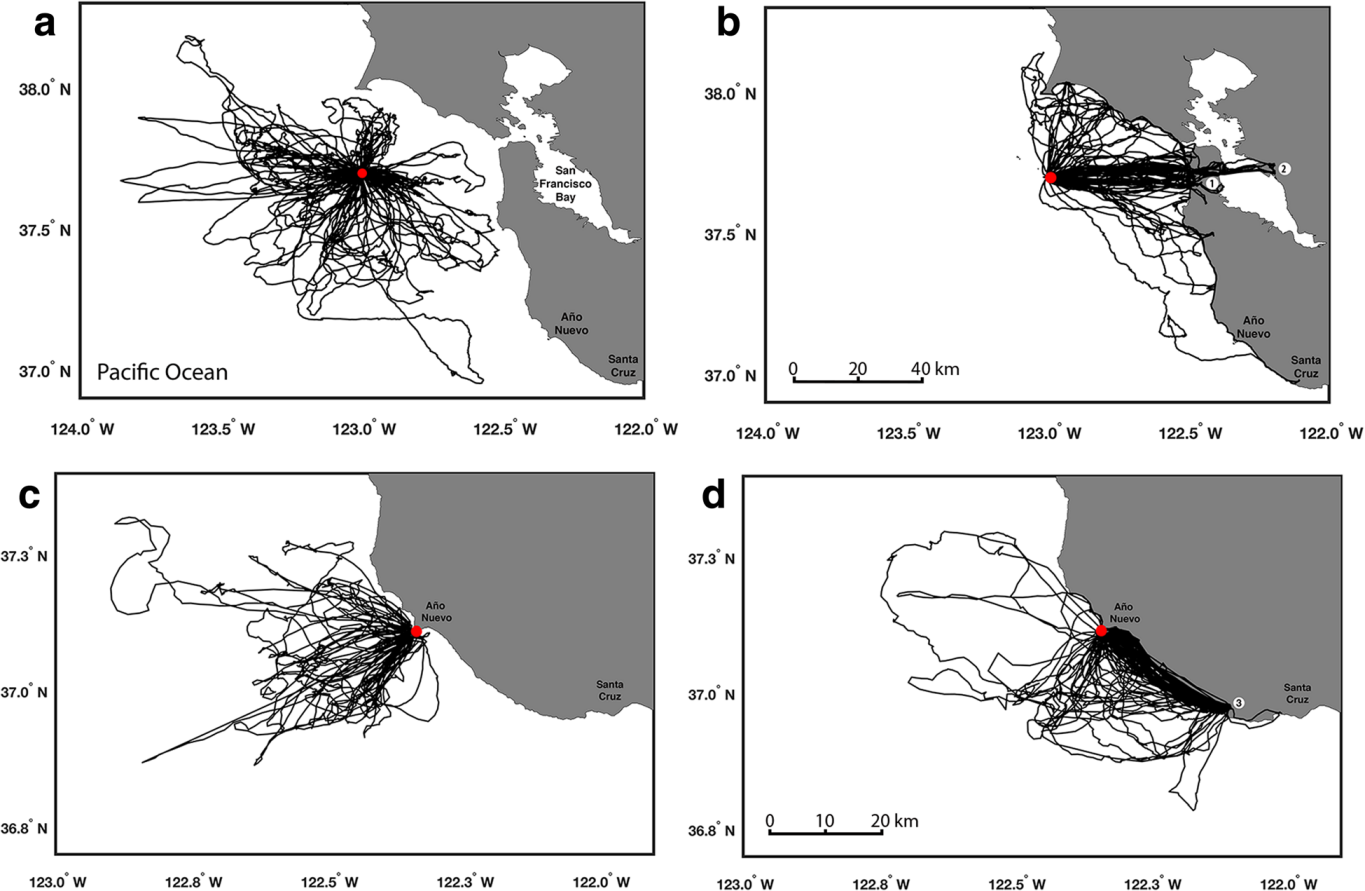
Tracklines of 61 western gulls (*Larus occidentalis*) equipped with GPS loggers at two central California colonies. **a** oceanic trips (*n* = 90) by gulls from Southeast Farallon Island (SFI); **b** trips (*n* = 41) to the coast and urban centers by gulls from SFI; **c** oceanic trips (*n* = 42) by gulls from Año Nuevo Island (ANI); and **d** trips (*n* = 103) to the coast by gulls from ANI. Red dots delineate the breeding colonies and white dots highlight landfills or food recycling centers visited by the gulls. 1) *Recology* in South San Francisco, 2) *Waste Management* in Oakland, and 3) *City of Santa Cruz Resource Recovery Facility*

Western gulls exhibited substantial variability in foraging trips within and between colonies and differing associations with habitats visited. Gulls from SFI traveled in all directions away from the colony and foraged up to 130 km from the colony (Figs. 1a & b). In addition, foraging behavior differed for trips in oceanic waters compared with trips over land. For example, the duration of trips were 3–4 times longer, and both maximum range and total distances traveled more than doubled when gulls traveled to land compared with ocean trips around the colony (Table 1). In contrast, gulls from ANI traveled predominantly southeast or west from their breeding colony (Figs. 1c & d) and trip durations, maximum ranges, and total distances traveled were similar regardless of whether gulls foraged at sea or over land (Table 1).

**Table 1:** Foraging parameters of GPS tracked western gulls from Southeast Farallon Island (SFI) and Año Nuevo Island (ANI)

Gull population	Trip type	Trips (n)	Trip duration (h)	Max range (km)	Distance traveled (km)	% of trip over land
SFI	Ocean	90	3.8 ± 3.3^a,b^	27.1 ± 20.3^a^	70.7 ± 58.9^a^	0
	Land	41	12.9 ± 7.5^a,c^	53.4 ± 15.5^a,c^	150.9 ± 59.3^a,c^	52.0 ± 22.6^c^
ANI	Ocean	42	2.9 ± 2.7^b^	20.6 ± 12.1	48.5 ± 31.4	0
	Land	103	3.6 ± 2.4^c^	20.8 ± 9.4^c^	51.2 ± 31.3^c^	73.4 ± 27.8^c^

Shown are means ± SD for trip duration, maximum range (i.e. distance) from breeding colony, total distance traveled, and percentage of trip over land (i.e. % of trip duration). For each population, trips are separated by whether gulls foraged in oceanic waters exclusively or whether they visited locations on land. See Methods for details on statistical treatments of the data

Significant statistical differences (*P* < 0.05) between trip types (ocean vs land) within a population (a; SFI only) and between populations within a trip type (b & c)

Population-level comparison of trip patterns revealed that 69% of all trips conducted by SFI gulls were to sea whereas only 29% of all trips by ANI gulls were to sea (Table 1). Oceanic trips for SFI birds also were significantly longer in duration, but not statistically greater in maximum range or total distance traveled (Table 1). Overwhelmingly, gulls from ANI foraged over land more frequently (71% of all trips) but durations were 3 times shorter, twice as close, and travel distances were 3 times less than land trips conducted by gulls from SFI. Gulls from ANI (1 km to closest mainland landfall) spent a greater proportion of their ‘land’ trip durations foraging on land (73.4%) compared with gulls at SFI (32 km to closest mainland landfall) where 52% of their ‘land’ trips were spent on land (Table 1).

**Fig. 2:**
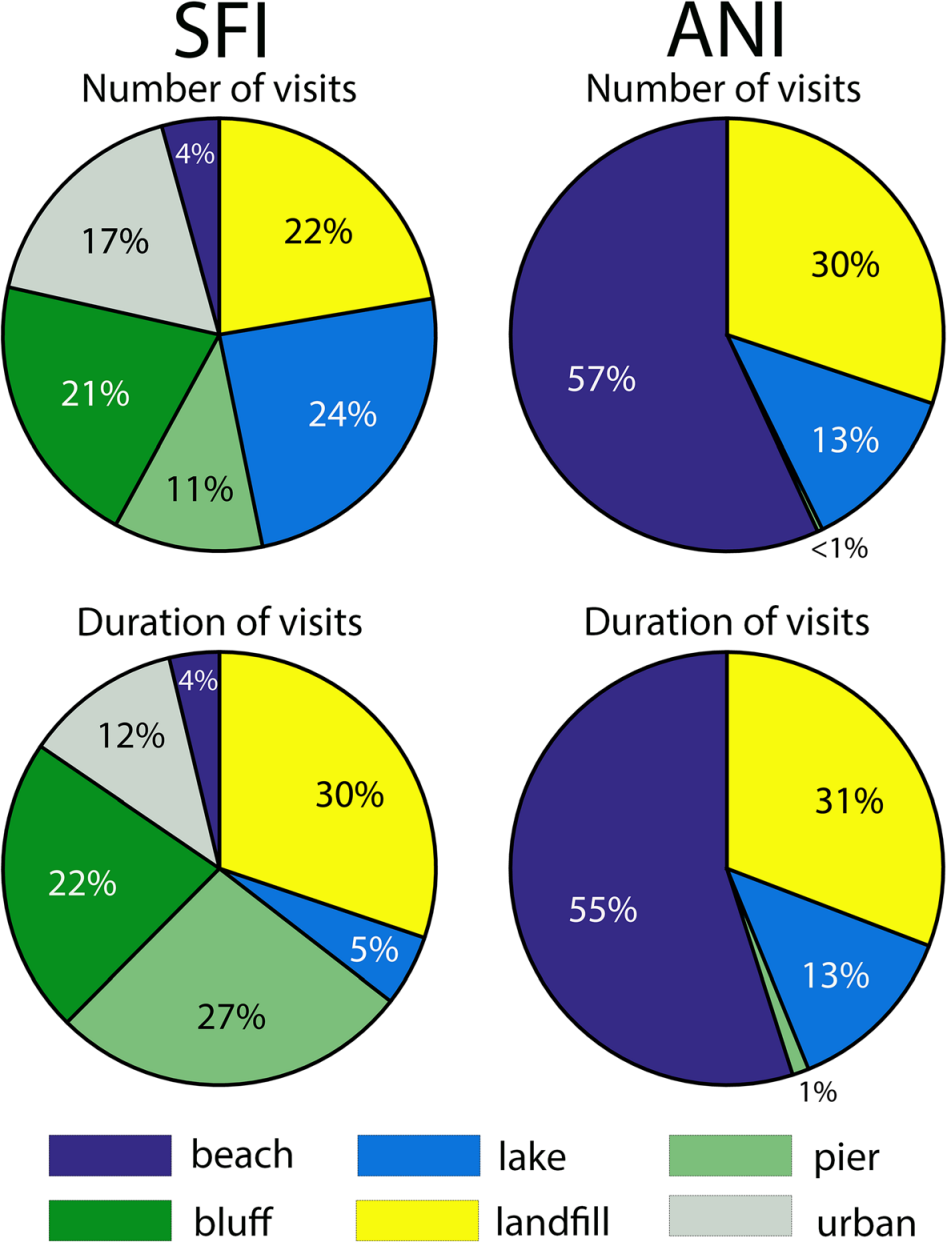
Proportions of land-based habitats visited by GPS tracked western gulls from populations at Southeast Farallon Island (SFI) and Año Nuevo Island (ANI). Shown are six different habitats determined using Google Earth and identified from stationary events of tracked gulls (see Methods). The top pie charts show population-level differences by numbers of visits to each habitat (*n* = 233 total visits for SFI and *n* = 262 total visits for ANI). The bottom pie charts show differences by duration of time spent at each habitat (*n* = 223.4 total hours for SFI and *n* = 238.8 total hours for ANI)

Gulls from SFI visited a greater variety of habitats while foraging on land compared to gulls from ANI (6 vs. 4, respectively). SFI gulls visited little beach habitat but made the greatest number of visits and spent the most time in urban habitats (74%), including piers, buildings, schools, and a lake within an urban park (Fig. 2). In contrast, ANI is located along a rural portion of the California coastline so there were essentially no visits to urban areas except one gull that visited a pier in Santa Cruz (Fig. 2). Otherwise, gulls from ANI visited a single landfill (30% of all visits and 31% of total duration at the City of Santa Cruz Resource Recovery Facility) and several beaches (57%) located between the landfill (south) and colony (north). In every case, gulls visited beach habitats while traveling to or returning from the landfill and this habitat had the greatest proportion of visits and time spent (Fig. 2).

### Activity specific behaviors and time activity budgets

**Fig. 3:**
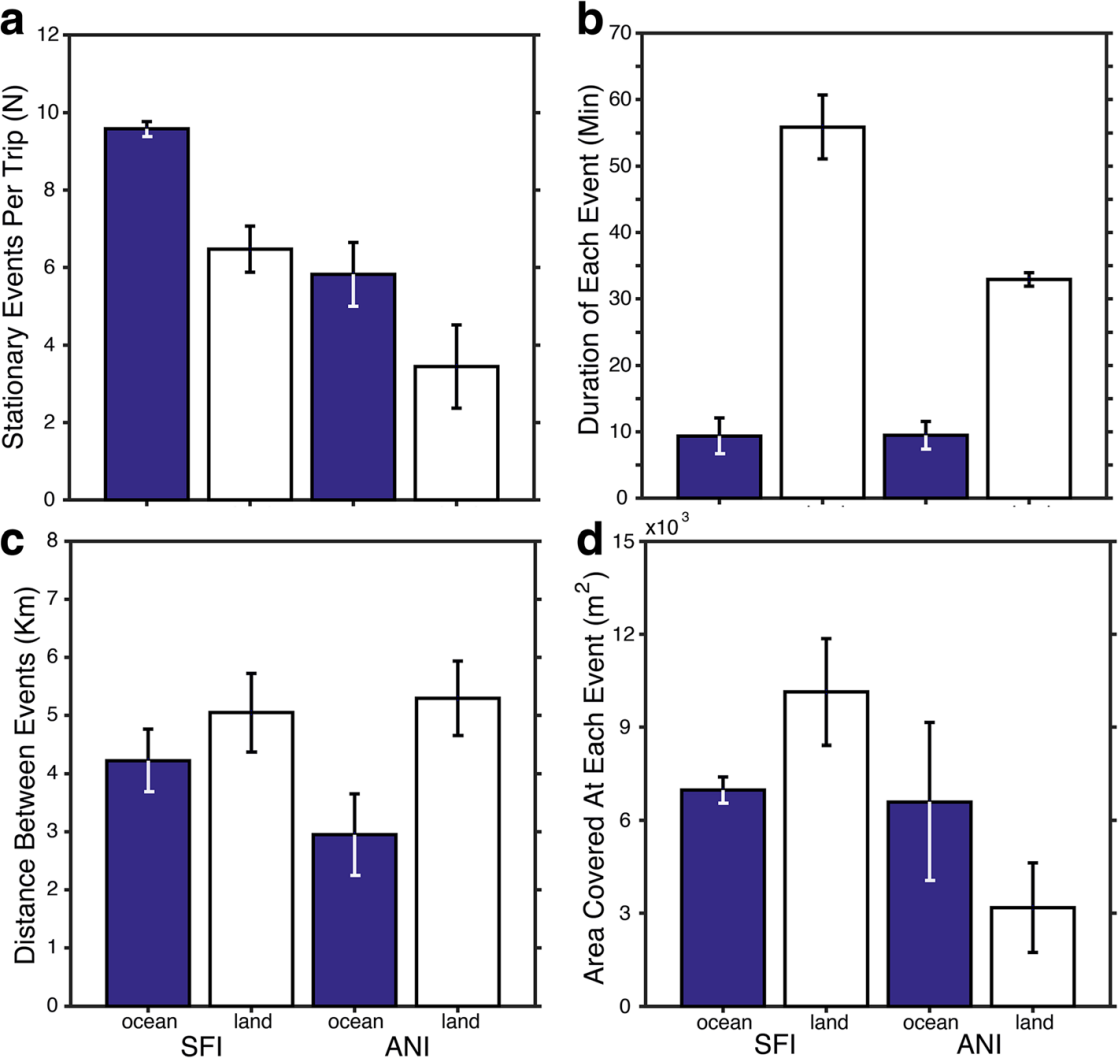
Foraging activity in relation to the mean (**a**) number of stationary events, (**b**) duration of time at each stationary event, (**c**) distance between stationary event locations, and (**d**) the spatial area of each stationary event location that was visited by GPS tracked western gulls from Southeast Farallon Island (SFI) and Año Nuevo Island (ANI). Activity was differentiated by habitat type for each foraging trip (see Methods). Shown are means ± SE and statistical results are reported in the text. In brief, the effect of habitat type (ocean or land) was significantly different within each population and the differences between populations were significant for time at each stationary event and the area size of each site where gulls were stationary for at least 5 min in an area within 0.5 km radius

Using our criteria to characterize stationary events, gulls clearly frequented a number of site locations on land (*n* = 33 sites) with repeated visits by the same gull and/or by others from the same breeding colony. In contrast, gulls from either population exhibited little repeatability of specific site locations when foraging at sea. Nevertheless, the number of stationary events was significantly greater during ocean trips (*F*_1,209_ = 8.73, *P* = 0.003) compared to land trips (Fig. 3a), and although the effect of colony origin was not significant, the interaction between habitat type and colony was statistically significant (*F*_1,209_ = 3.94, *P* = 0.048). Even though the number of stationary events by gulls was greater during ocean trips, mean duration on the water was about 10 min for gulls from both colonies compared with mean durations of 30 and 60 min during land trips for gulls from ANI and SFI, respectively (Fig. 3b). Both habitat type and colony origin significantly influenced the mean duration of time a gull remained at the site of a stationary event (*F*_1,232_ = 221, *P* < < 0.001 and *F*_1,232_ = 7.94, *P* = 0.005, respectively). Habitat type (but not colony origin) also significantly affected the distance gulls traveled between stationary events (*F*_1,207_ = 21.3, *P* < < 0.001) where the mean distance while traveling over land was approximately 5 km between stationary event locations compared with 3–4 km during ocean trips (Fig. 3c). The area searched or covered by gulls when stationary was greatest (10,139 ± 1720 m^2^) for trips to land by gulls from SFI and least (3178 ± 1446 m^2^) for trips to land by gulls from ANI (Fig. 3d). Ultimately, the area of each stationary event location was influenced by habitat type and colony origin (*F*_1,231_ = 13.9, *P* < 0.001 and *F*_1,231_ = 9.67, *P* = 0.002, respectively).

**Fig. 4:**
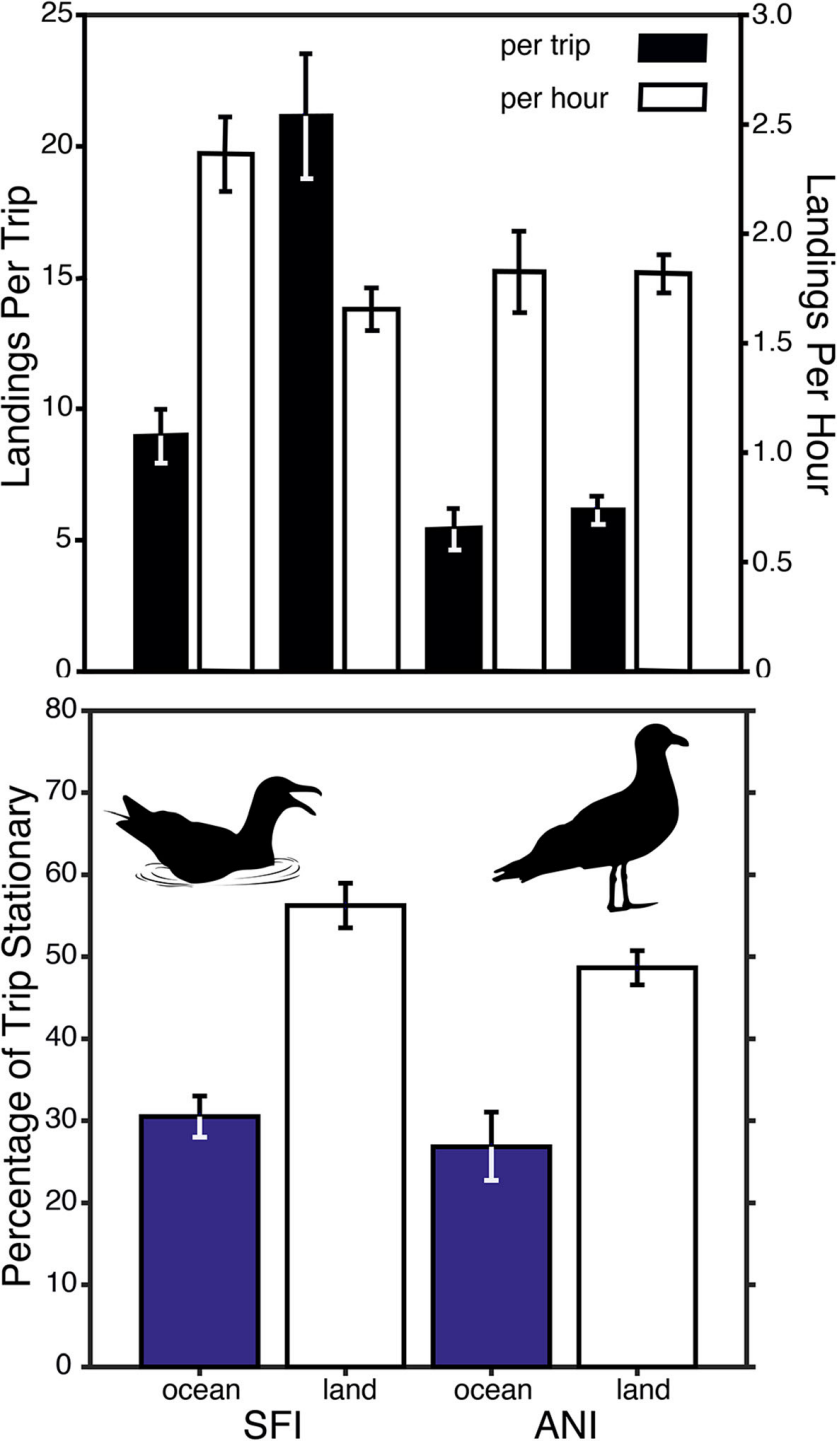
Behavioral patterns of tracked western gulls from Southeast Farallon Island (SFI) and Año Nuevo Island (ANI). The top pane shows the number of landings when foraging at sea or on land. The bottom pane shows the percentage of time gulls were stationary (< 6 km h^−1^) when foraging at sea or on land. Shown are means ± SE

We also characterized the frequency and rate of individual landings/take offs based on changes in travel speeds (Additional file 2: Figure S1). The number of landings/take offs per trip was greatest for gulls that traveled over land from SFI (21.1 ± 2.4 landings/take offs per trip) but gulls conducting ocean trips from SFI had the highest rate of landings/take offs (2.4 ± 0.2 landings per hour; Fig. 4). The number of landings/take offs per trip and per hour for gulls from ANI were essentially identical regardless of whether gulls traveled to sea or land. For both landings/take offs per trip and per hour, the differences between populations and habitat type/colony interactions were statistically significant (*F*_1,260_ > 5.5, *P* < 0.02 for comparison of landings/take offs per trip and per hour). Finally, trip type (land or sea) profoundly affected the percentage of time that gulls remained stationary versus transiting. During land trips from either colony, gulls were stationary for approximately 50–55% of their trip compared with 25–30% when conducting an ocean trip (*F*_1,261_ = 63.8, *P* < < 0.001; Fig. 4). Gulls from SFI also had significantly greater proportions of time spent stationary while at sea or on land compared with gulls from ANI (*F*_1,261_ = 7.22, *P* = 0.008).

**Fig. 5:**
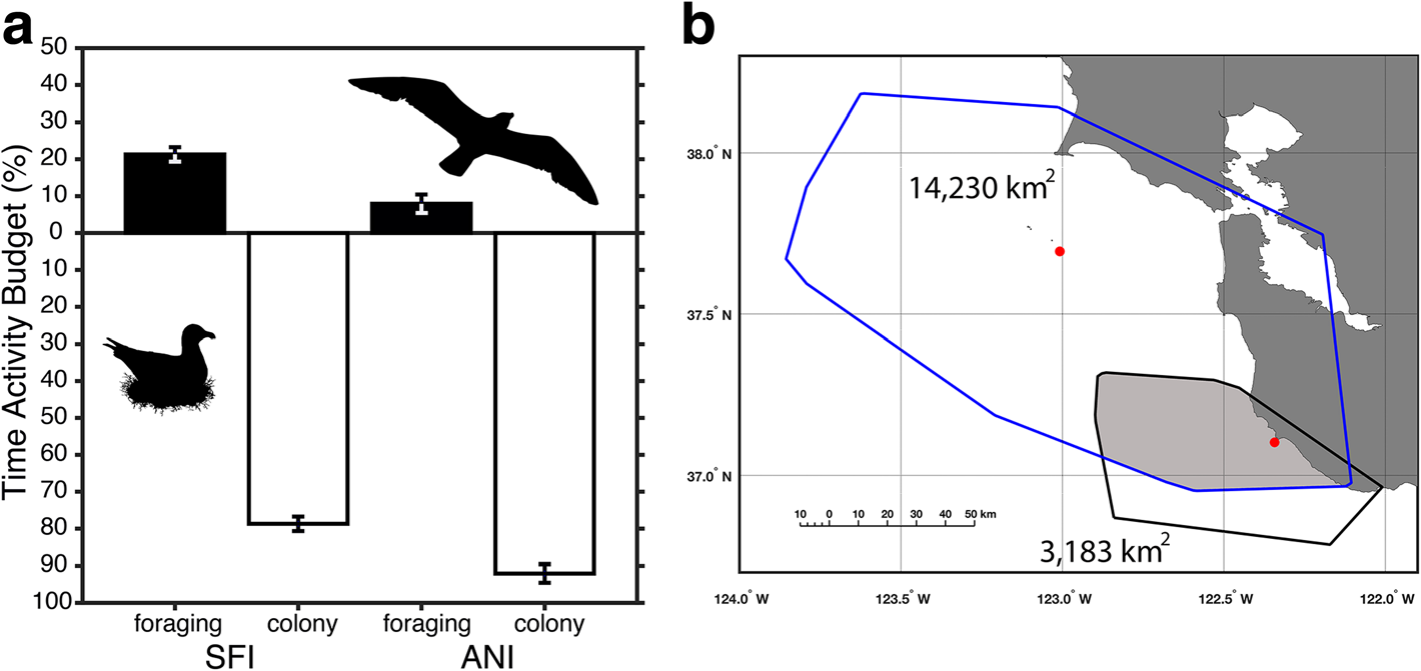
**a** Time activity budgets of GPS tracked western gulls from Southeast Farallon Island (SFI; *n* = 40) and Año Nuevo Island (ANI, *n* = 21). Gulls were either away from the nest foraging (black bars) or in the breeding colony incubating eggs (white bars) during the study period. Shown are means ± SE where population-level differences for each activity were significantly different (statistics are reported in Results). **b** Population-level home ranges of gulls from SFI (blue line) and ANI (black line) determined using minimum convex polygons around the GPS tracklines. Red dots mark the locations of each breeding colony and the shaded region represents the overlap (2013 km^2^) between home ranges for each population

A central aspect of the current project focused on whether gull activity budgets differed between colonies. This metric combined the duration of time that each gull spent away from its nest foraging over consecutive trips versus the cumulative time spent at the nest incubating or protecting its brood between foraging trips. Overall, gulls from SFI were away 3 times longer (21%) than gulls from ANI (*F*_1,59_ = 16.8, *P* < 0.001; Fig. 5a) and thus, were present in the colony 13% less than gulls from ANI. These differences were consistent with variation in population-level home ranges where gulls from SFI utilized a combined area of 14,230 km^2^, which was 4.5 times larger than the home range of gulls from ANIS (Fig. 5b).

### Fidelity index

**Fig. 6:**
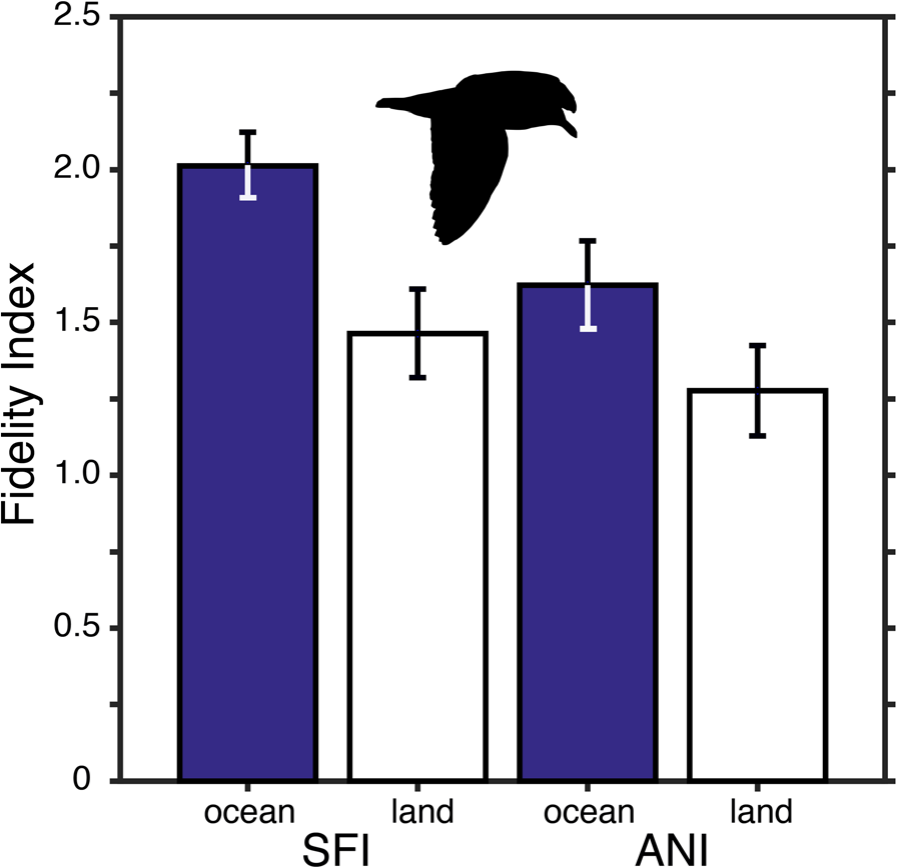
Fidelity Index (FI) of foraging habitat by gull population where a lower index (range 0 to 4) indicates greater fidelity of gulls to forage at similar destinations (either repeated trips by the same individual and/or within a population). Gulls from each population exhibited greater fidelity to foraging locations on land compared to locations at sea (e.g. Fig. 1). Shown are means ± SE where FI was statistically different between habitat types and populations (see Results for statistics)

Overall, fidelity index varied significantly with habitat type (*F*_1,212_ = 4.72, *P* = 0.031) and colony (*F*_1,212_ = 4.16, *P* = 0.042). The greatest fidelity index (i.e. low site fidelity) was established for oceanic trips of gulls from SFI (Fig. 6), consistent with tracklines that radiated away from the beeding colony in all directions (Fig. 1a). Essentially, there was little directional bias in the maximum foraging ranges of gulls from SFI. In contrast, the least fidelity index (i.e. high site fidelity) was established for gulls from ANI that traveled to sites on land (Fig. 6), consistent with the narrow directional bias exhibited by gulls traveling southeast from their colony at ANI to the landfill in Santa Cruz (Fig. 1d).

## Discussion

Our study compared the foraging movements of tracked western gulls from two populations that differed in their access to terrestrial food resources. Gulls from SFI, located more than 30 km from the nearest coastline, conducted 68% of all foraging trips in oceanic waters around the breeding colony. In contrast, gulls from ANI conducted 71% of all foraging trips to terrestrial sites along the coast and overwhelmingly to a particular landfill southeast of the colony (Fig. 1d). Thus, habitat use differed significantly between gull populations, and this influenced their foraging behavior and activity patterns.

Compelling evidence from an examination of western gull diets demonstrated that individuals often specialize their foraging strategies when breeding [14, 15, 48]. We did not quantify individual specialization (e.g. [49]) because the typical durations of our tracking effort were only 5 to 8 days per gull per season. Longer tag deployments would likely be required to capture niche widths of individuals for comparison with the population (reviewed in [3]). Nevertheless, we did observe some patterns that indicate specialization among individuals from each gull population. For example, 28 of 61 gulls used a mixed strategy where they alternated (not necessarily on consecutive trips) foraging trips between marine and terrestrial habitats (Table 2). The remaining gulls either foraged exclusively at sea (17 of 61) or on land (16 of 61) over multiple trips. Only one gull from ANI foraged exclusively at sea on every trip and 7 of 16 gulls that foraged only on land were from SFI. As indicative as these patterns may be, more research is needed to better characterize individual specialization based on specific foraging behaviors and habitat use.

**Table 2:** Numbers and proportions of western gull foraging behavior by gull numbers and by numbers of trips

Trip type	Number of gulls	% of gulls	Number of Trips	% of trips
Ocean only	17	27.9	60	21.7
Land only	16	26.2	44	16.0
Mixed	28	45.9	172	62.3
Totals	61	100	276	100

For gulls that exhibited a mixed foraging strategy, 72 trips were at sea and 100 trips were to sites on land

Although we did not characterize individual foraging behaviors *per se*, we examined behavioral plasticity within and between gull populations based on patterns of habitats visited and used (e.g. Fig. 2). Both gull populations exhibited differences in activity patterns associated with foraging habitat. For example, when foraging at sea, gulls from both populations visited a greater variety of locations (areas of stationary events), but spent less time sitting on the sea surface (i.e. feeding or resting) at each site, and overall, a greater percentage of time traveling. The random nature of travel directions and the lack of repeatability in travel destinations combined, all indicate that gulls probably are increasing the number of feeding opportunities at sea by maximizing the total area searched, rather than commuting to a specific, predictable location [50]. This is consistent with the generality and large breadth recorded among western gull diets [14, 15, 37]. Furthermore, our tracking results reflect similarities with at-sea foraging patterns of gulls measured in several other studies in the Atlantic [20, 21, 26, 27].

In contrast with the activity patterns and randomness of foraging at sea, western gulls traveling to land regularly used similar travel routes and generally made repeated visits to specific locations on land (especially gulls from ANI, Figs. 1d & 2). Gulls from SFI, however, had to travel farther to reach landfall resulting in longer foraging trips overall. Foraging theory generally predicts that longer travel times to a food source requires a longer stay at the source to make foraging effort profitable, especially when foraging from a central place [1, 51, 52]. Gulls from SFI followed this pattern where they visited a greater variety of locations on land and remained for longer durations at each location (Figs. 2 & 3b), compared with the activity patterns of gulls from ANI. Although distances between stationary events on land were not dramatically different between populations, the area covered (i.e. potentially searched) during each stationary event was greatest for the sites visited by gulls from SFI. Repeated visits to locations more distant from their breeding colony indicates some level of predictability and/or profitability for SFI gulls to recover travel costs. Unfortunately, this remains untested because we were not able to measure body mass changes after each foraging trip without causing too much colony disturbance.

Several land locations were visited repeatedly by gulls across study years but many appeared to be for activities like resting, bathing, etc. because there was no discernable food source (e.g. parking lot, bluffs, beaches with river outflow, buildings). However, there were three discrete land locations that gulls routinely exploited as a food source including two landfills and a food recycling center (Fig. 1b, d & 2, Additional file 4: Figure S3, Additional file 5: Figure S4, Additional file 6: Figure S5). Landfills, agriculture fields, aquaculture farms, and food processing factories have all been shown to be viable resources for gulls [20, 21, 33, 53, 54] and it may explain their ability to buffer environmental variability and minimize competition [9, 27].

A key element of our study compared population-level plasticity in foraging behavior and its association with colony size. Numerous studies support the theory of Storer-Ashmole’s halo [55, 56] where resource depletion around a breeding colony co-varies with colony size, thus forcing birds to range farther to find food ([57–60], others). Although we did not specifically test this theory, the general patterns in foraging ecology of western gulls that we observed (Fig. 1a-d, Table 1) support Storer-Ashmole’s halo because SFI has an island population of seabirds several orders of magnitude greater than ANI [41]. Furthermore, in addition to ranging farther from the breeding colony, western gulls from SFI also foraged over a larger expanse of marine and terrestrial habitats (Fig. 5b) with lower site fidelity, but with greater access to diverse habitats (e.g. oceanic, coastal, estuarine, freshwater lakes, large inshore embayments, and dense urban areas). These observations support the hypothesis that gulls from SFI have broader niches than gulls at ANI, which could be a mechanism to reduce intra-specific competition through spatial segregation (reviewed in [3] and supported by [5, 9, 20, 61–63]).

The present study focused on the foraging movements of breeding western gulls when parents alternate between foraging and incubating eggs. Aggression and egg cannibalism by neighboring gulls can greatly alter reproductive success, especially when a single parent is left to protect the brood (normal clutch of 3 eggs; [64–66]). Since 1990, annual productivity of western gulls at SFI has been less than one chick fledged per pair, which is unsustainable [41]. In contrast, gulls at ANI fledge an average of 1.23 ± 0.08 chicks per pair (long-term average from 1999 to 2015; [42]). We hypothesize that the population-level foraging behavior we observed contributes to the differences in colony-specific breeding success. Because gulls at SFI conduct longer foraging trips, time activity budgets for each population indicate that tracked individuals from SFI were absent from the nest 22% (present 78%) of the time compared with 7% (present 93%) for gulls at ANI. Thus, a greater absence from the nest, combined with larger breeding colony size may contribute to lower productivity at SFI through enhanced predation risk and/or greater costs associated with nest vigilance.

## Conclusions

Our results reveal substantial differences in foraging behavior and habitat use between two populations of western gulls in central California, with implications for explaining differences in breeding success at each colony. The generality of these patterns for other populations of western gulls to the south (e.g. Mexico and southern California) and to the north (Oregon) of our study colonies is unknown. However, studies are underway that will attempt to address such variation in movement strategies in the future. This research will be informative because the other colonies vary in both population sizes and their proximity to coastal resources.

The population level plasticity shown in our study may be a key factor that allows western gulls to adapt to changing conditions. California currently is the most populous state in the United States, leading to greater impacts on coastal resources through urban development and greater extraction of natural resources [67]. Thus, with a more complete characterization of individual−/population-level plasticity, monitoring the ecology and population dynamics of western gulls may offer unique insight into the resiliency of a conspicuous marine vertebrate [22] and provide guidance for future development of renewable energy sources in ocean habitats [23–25].

## Additional files

Additional file 1: Table S1. Summary of GPS tag deployments and recoveries by year (DOCX 73 kb)

Additional file 2: Figure S1. A) histogram of travel speeds of GPS tracked western gulls used to establish locations (in B &C) where gulls were stationary based on travel speeds less than 6 km h^−1^. B) GPS track lines (black lines) and stationary locations (red dots) of western gulls from Southeast Farallon Island and Año Nuevo Island, off central California, USA. C) shows the landing locations without tracklines. All dots are the same size and thus do not reflect area or intensity of habitat use (PNG 405 kb)

Additional file 3: Figure S2. A) track line (black) and GPS locations (blue dots) of a western gull from Southeast Farallon Island. Yellow dots along the track line were locations where the gull was stationary (travel speed <6 km h^−1^). Arrows mark the direction of travel from the colony or mainland. B) zoomed images of the track line over land where stopover sites were differentiated from brief stops by identifying consecutive stationary locations within a 0.5 km radius for longer than 5 min. For each stopover site, we calculated the duration, area (in sq. meters), and distance between sites. The enlargements show how two sites were quantified using minimum convex polygons. In this case, the bird made two separate searches in roughly the same general location on the same trip but separated by greater than 5 min (PNG 662 kb)

Additional file 4: Figure S3. A) Movement patterns of GPS tracked western gulls within the city of San Francisco, California. B) is an enlargement of *Recology*, a business that recycles food scraps and was a frequent stop over site for gulls traveling to the city from Southeast Farallon Island (PNG 6414 kb)

Additional file 5: Figure S4. Movement patterns of GPS tracked western gulls from Southeast Farallon Island overlaid onto satellite imagery of the city of Oakland, California. *Waste Management* was a resource recovery center frequented by the gulls (PNG 1402 kb)

Additional file 6: Figure S5. A) Movement patterns of GPS tracked western gulls from Año Nuevo Island overlaid onto satellite imagery of the Santa Cruz coastline. Panels B & C are enlargements of the City of Santa Cruz Resource Recovery Facility (Santa Cruz, California) frequented by 19 of 20 gulls tracked from the colony. This site was also the most common site visited by all tracked gulls with 80 visitations (PNG 5862 kb)

## Abbreviations

ANI: Año Nuevo Island
Cm: centimeters
G: grams
Hrs: hours
Km: kilometers
M: meters
SD: Standard deviation
SE: Standard error
SFI: Southeast Farallon Island
