# Supplementary material for: Population-level plasticity in foraging behavior of western gulls (Larus occidentalis)
Source: Mov Ecol. 2017 Dec 19;5:27. doi: 10.1186/s40462-017-0118-9 (PMC5735870; doi:10.1186/s40462-017-0118-9)
Supplement: Supplementary file 1 — Summary of GPS tag deployments and recoveries by year (DOCX 73 kb) [file 40462_2017_118_MOESM1_ESM.docx]

**Table S1** Summary of western gulls tracked with GPS loggers at Southeast Farallon Island (SFI) and Año Nuevo Island (ANI), central California, USA. Shown are the numbers of gulls captured for logger deployment and recaptured for logger recovery. All but three gulls (one from SFI and two from ANI), continued breeding after the study dates. The percentages reflect the proportion of gulls not recaptured or GPS loggers not recovered from the number of gulls captured (or loggers deployed) in a given year. The percentage of GPS loggers yielding viable data were calculated from the number of loggers recovered.

|  |  |  | Gulls studied | | GPS loggers | |  |
| --- | --- | --- | --- | --- | --- | --- | --- |
| colony | dates | year tracked | captured | recaptured | recovered | viable data | trips |
| **SFI** | 5/26-5/31 | 2013 | 15 | 11 (73%) | 8 (53%) | 8 (100%) | 13 |
|  | 6/01-6/12 | 2014 | 22 | 20 (91%) | 20 (91%) | 20 (100%) | 65 |
|  | 5/23-6/03 | 2015 | 15 | 15 (100%) | 15 (100%) | 15 (100%) | 53 |
| **ANI** | 5/20-6/17 | 2013 | 9 | 8 (89%) | 7 (78%) | 7 (100%) | 50 |
|  | 5/14-6/10 | 2014 | 12 | 9 (75%) | 6 (50%) | 4 (67%) | 8 |
|  | 5/08-6/09 | 2015 | 16 | 13 (81%) | 13 (81%) | 10 (77%) | 87 |
| **Totals** |  | **3** | **89** | **76 (85%)** | **69 (78%)** | **61 (88%)** | **276** |
