# Supplementary figures and images for: Population-level plasticity in foraging behavior of western gulls (Larus occidentalis)
Source: Mov Ecol. 2017 Dec 19;5:27. doi: 10.1186/s40462-017-0118-9 (PMC5735870; doi:10.1186/s40462-017-0118-9)

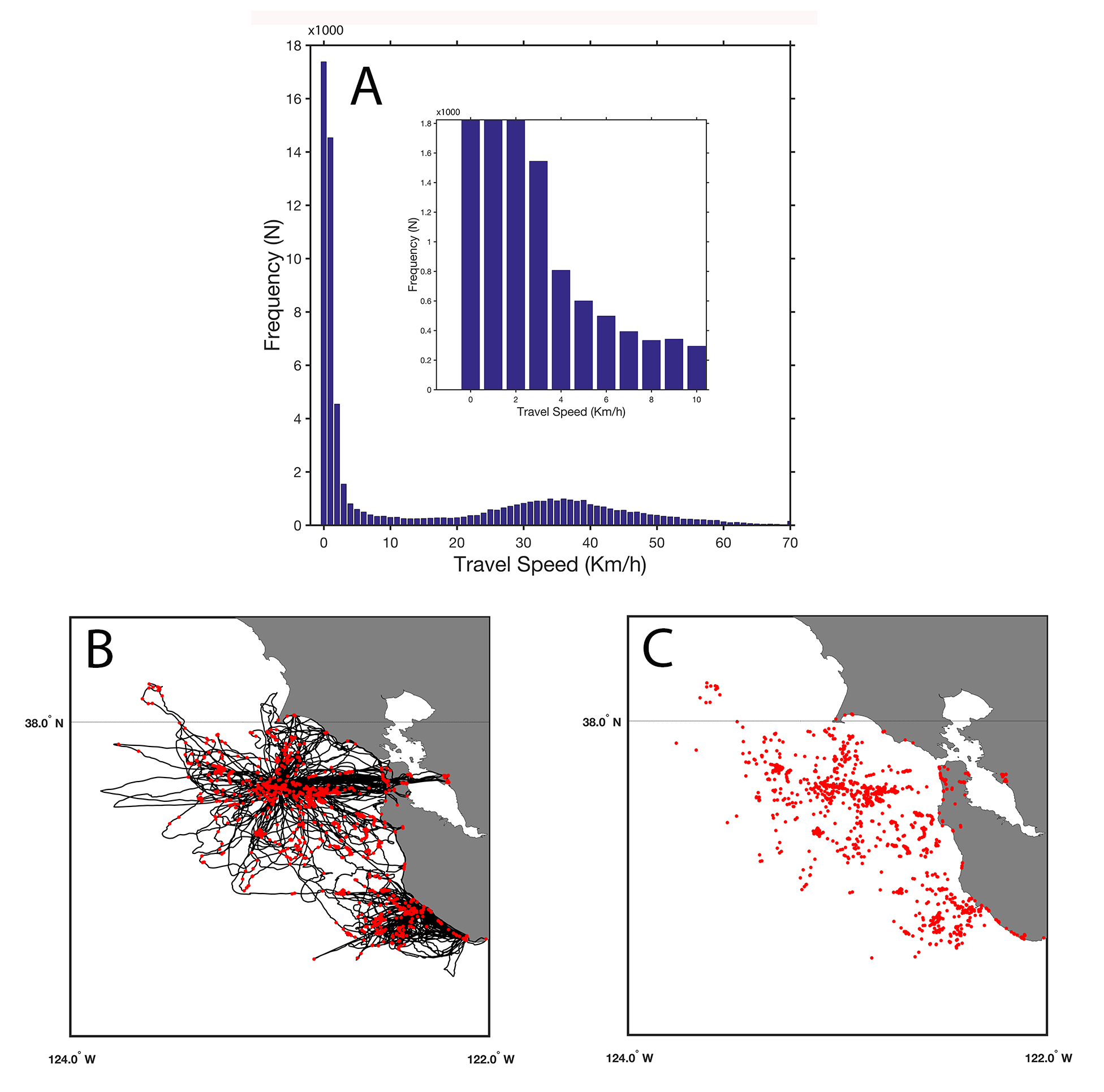

Supplement: Supplementary file 2 — A) histogram of travel speeds of GPS tracked western gulls used to establish locations (in B &C) where gulls were stationary based on travel speeds less than 6 km h−1. B) GPS track lines (black lines) and stationary locations (red dots) of western gulls from Southeast Farallon Island and Año Nuevo Island, off central California, USA. C) shows the landing locations without tracklines. All dots are the same size and thus do not reflect area or intensity of habitat use (PNG 405 kb) [file 40462_2017_118_MOESM2_ESM.png]

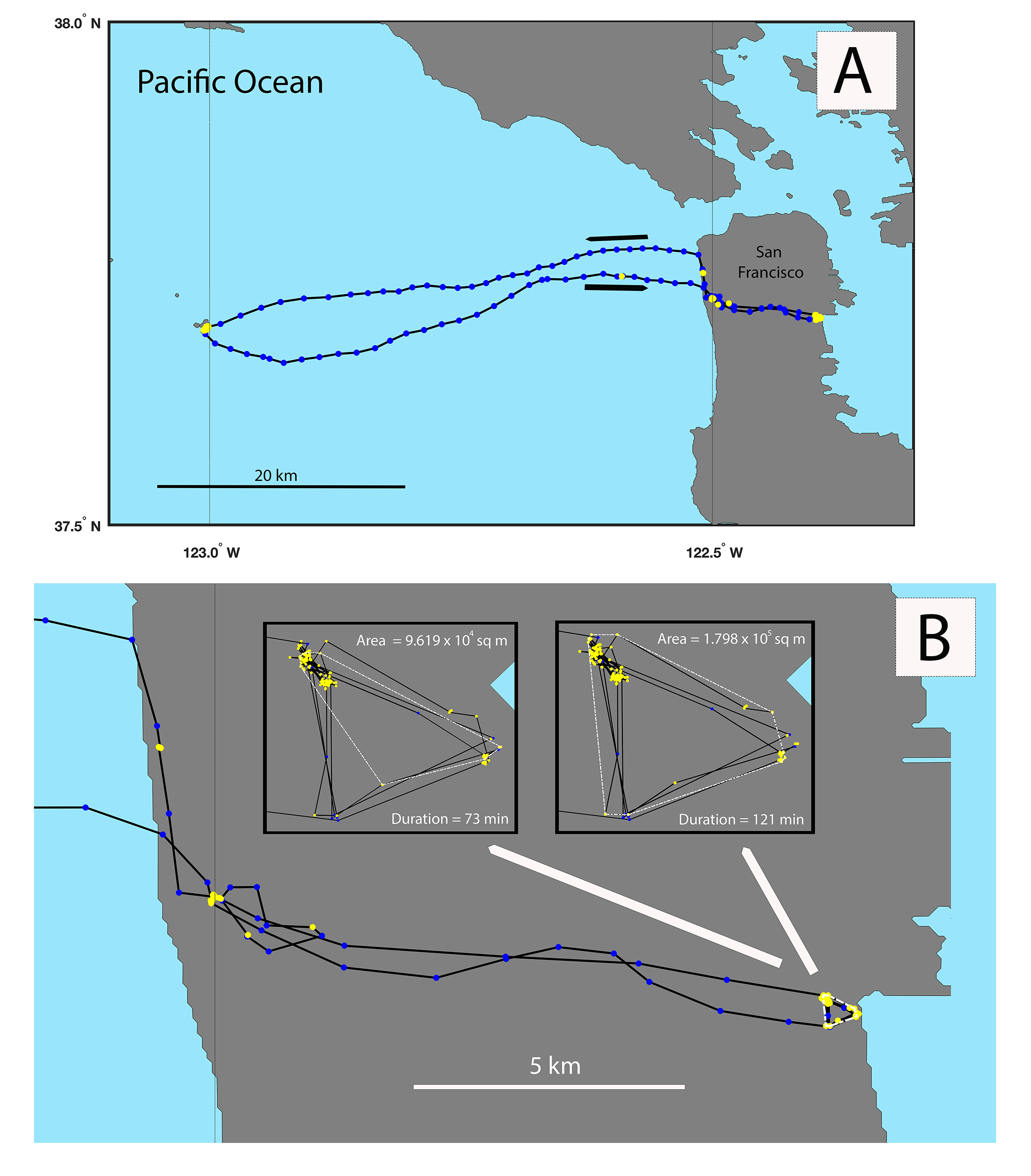

Supplement: Supplementary file 3 — A) track line (black) and GPS locations (blue dots) of a western gull from Southeast Farallon Island. Yellow dots along the track line were locations where the gull was stationary (travel speed <6 km h−1). Arrows mark the direction of travel from the colony or mainland. B) zoomed images of the track line over land where stopover sites were differentiated from brief stops by identifying consecutive stationary locations within a 0.5 km radius for longer than 5 min. For each stopover site, we calculated the duration, area (in sq. meters), and distance between sites. The enlargements show how two sites were quantified using minimum convex polygons. In this case, the bird made two separate searches in roughly the same general location on the same trip but separated by greater than 5 min (PNG 662 kb) [file 40462_2017_118_MOESM3_ESM.png]

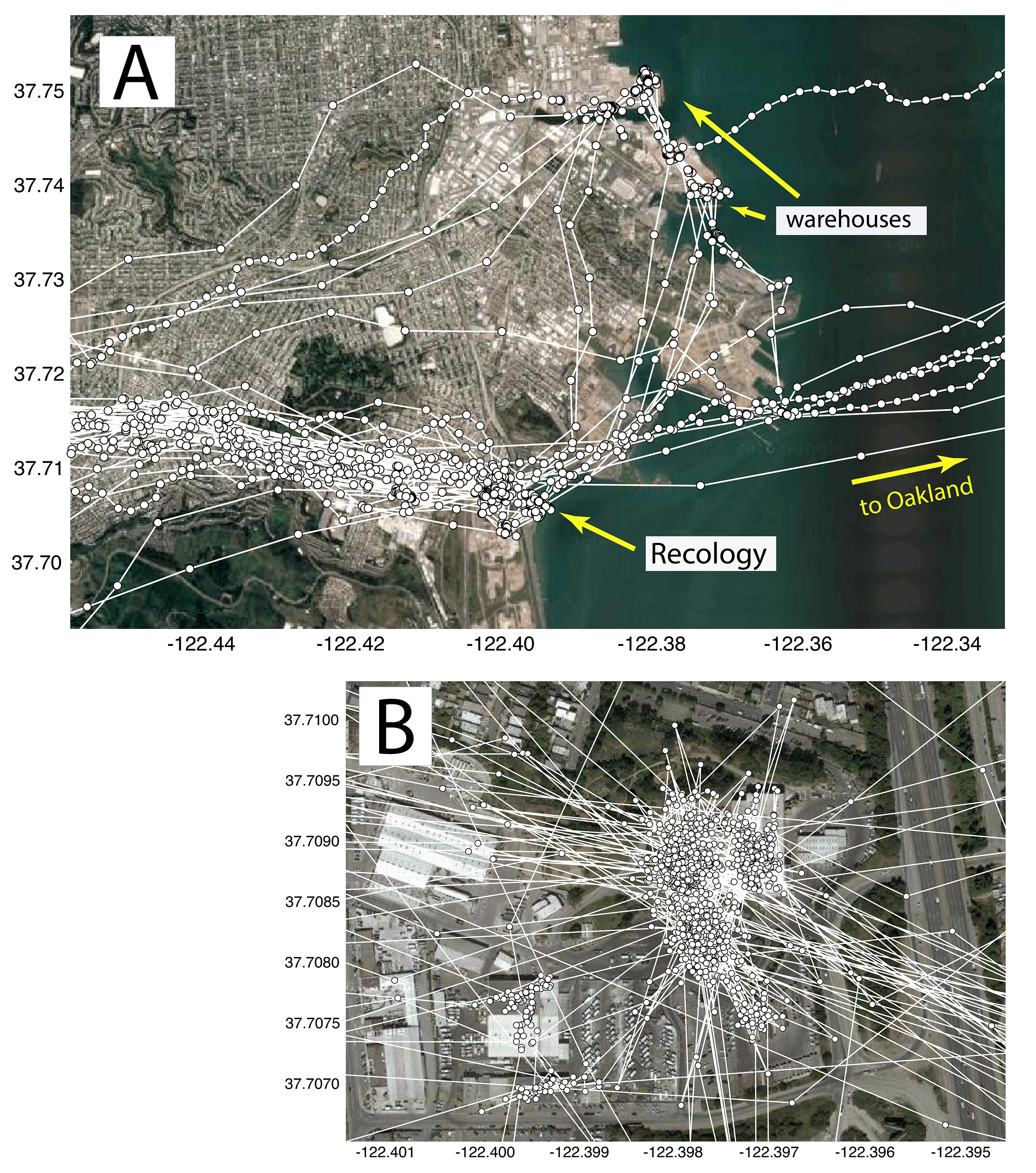

Supplement: Supplementary file 4 — A) Movement patterns of GPS tracked western gulls within the city of San Francisco, California. B) is an enlargement of Recology, a business that recycles food scraps and was a frequent stop over site for gulls traveling to the city from Southeast Farallon Island (PNG 6414 kb) [file 40462_2017_118_MOESM4_ESM.png]

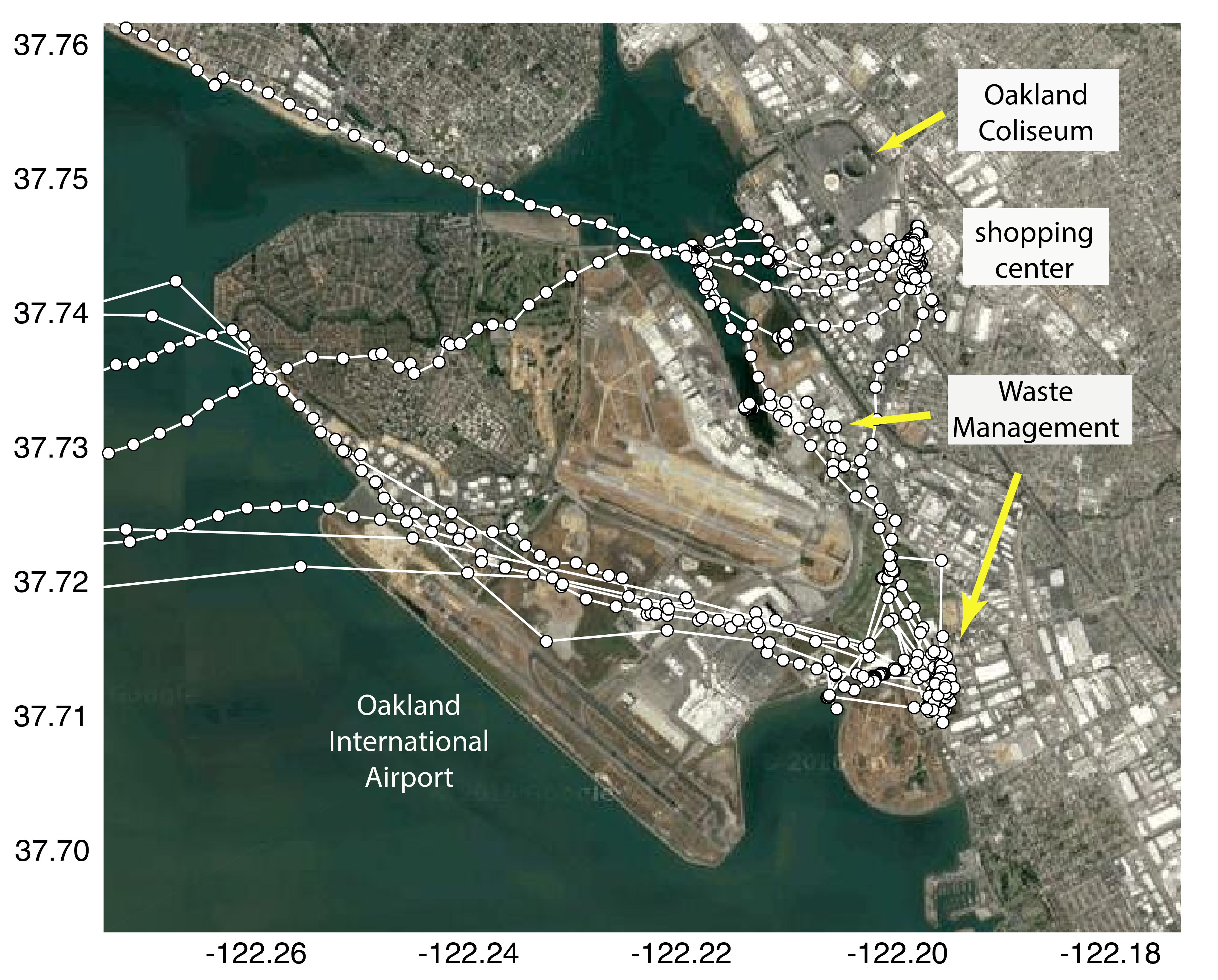

Supplement: Supplementary file 5 — Movement patterns of GPS tracked western gulls from Southeast Farallon Island overlaid onto satellite imagery of the city of Oakland, California. Waste Management was a resource recovery center frequented by the gulls (PNG 1402 kb) [file 40462_2017_118_MOESM5_ESM.png]

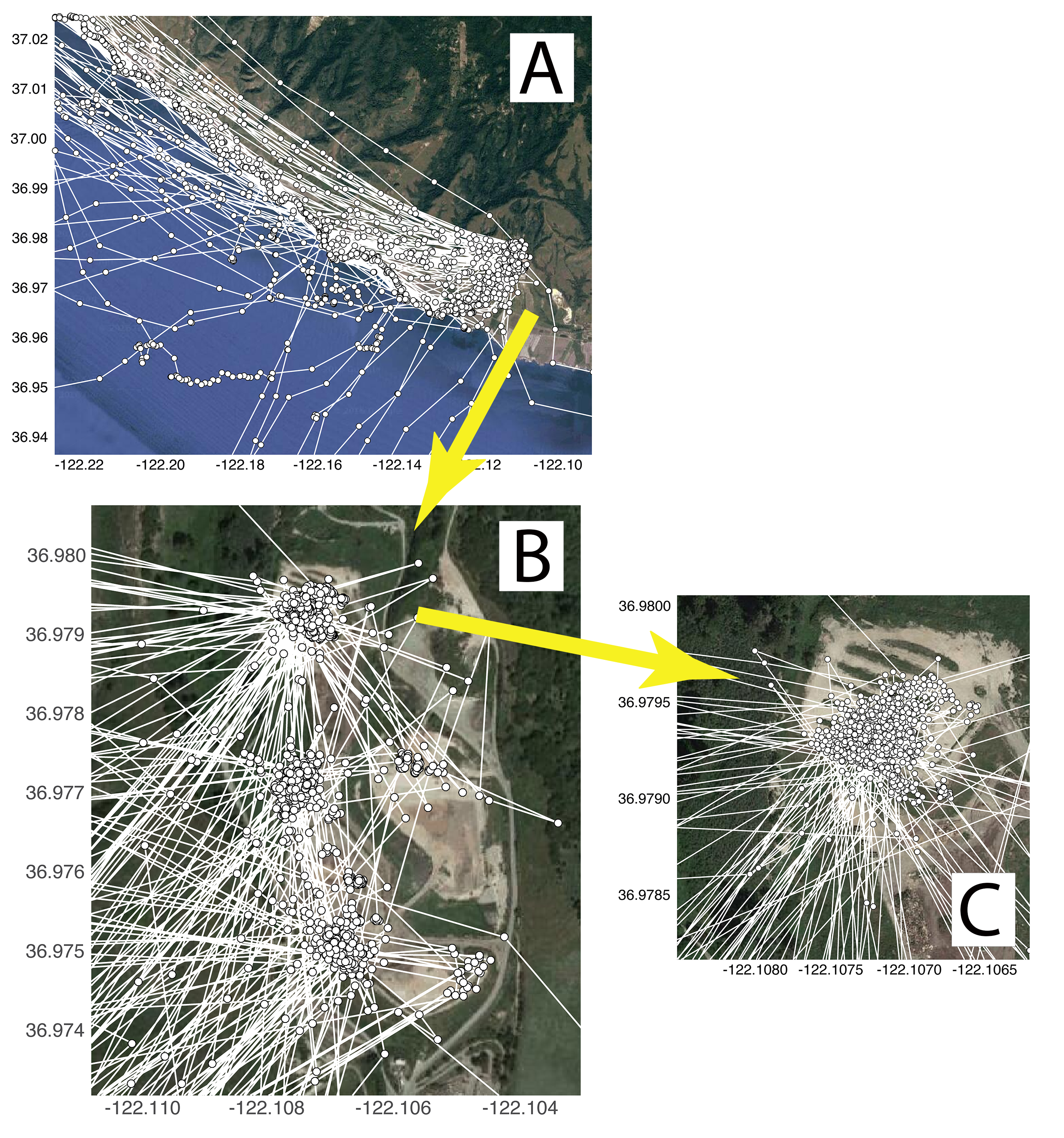

Supplement: Supplementary file 6 — A) Movement patterns of GPS tracked western gulls from Año Nuevo Island overlaid onto satellite imagery of the Santa Cruz coastline. Panels B & C are enlargements of the City of Santa Cruz Resource Recovery Facility (Santa Cruz, California) frequented by 19 of 20 gulls tracked from the colony. This site was also the most common site visited by all tracked gulls with 80 visitations (PNG 5862 kb) [file 40462_2017_118_MOESM6_ESM.png]
